# Tropine Forming Tropinone Reductase Gene from *Withania somnifera* (Ashwagandha): Biochemical Characteristics of the Recombinant Enzyme and Novel Physiological Overtones of Tissue-Wide Gene Expression Patterns

**DOI:** 10.1371/journal.pone.0074777

**Published:** 2013-09-25

**Authors:** Amit Kumar Kushwaha, Neelam Singh Sangwan, Prabodh Kumar Trivedi, Arvind Singh Negi, Laxminarain Misra, Rajender Singh Sangwan

**Affiliations:** 1 CSIR-Central Institute of Medicinal and Aromatic Plants (CSIR-CIMAP), Lucknow, Uttar Pradesh, India; 2 CSIR-National Botanical Research Institute (CSIR-NBRI), Rana Pratap Marg, Lucknow, Uttar Pradesh, India; 3 Bio-Processing Unit (*An Autonomous Institute under Department of Biotechnology, Govt. of India*), Interim Facility, C-127, Phase-8, Industrial Area, S.A.S. Nagar, Mohali, Punjab, India; University of Delhi South Campus, India

## Abstract

*Withania somnifera* is one of the most reputed medicinal plants of Indian systems of medicine synthesizing diverse types of secondary metabolites such as withanolides, alkaloids, withanamides etc. Present study comprises cloning and *E. coli* over-expression of a tropinone reductase gene (*WsTR-I*) from *W. somnifera*, and elucidation of biochemical characteristics and physiological role of tropinone reductase enzyme in tropane alkaloid biosynthesis in aerial tissues of the plant. The recombinant enzyme was demonstrated to catalyze NADPH-dependent tropinone to tropine conversion step in tropane metabolism, through TLC, GC and GC-MS-MS analyses of the reaction product. The functionally active homodimeric ∼60 kDa enzyme catalyzed the reaction in reversible manner at optimum pH 6.7. Catalytic kinetics of the enzyme favoured its forward reaction (tropine formation). Comparative 3-D models of landscape of the enzyme active site contours and tropinone binding site were also developed. Tissue-wide and ontogenic stage-wise assessment of *WsTR-I* transcript levels revealed constitutive expression of the gene with relatively lower abundance in berries and young leaves. The tissue profiles of *WsTR-I* expression matched those of tropine levels. The data suggest that, in *W. somnifera*, aerial tissues as well possess tropane alkaloid biosynthetic competence. *In vivo* feeding of U-[^14^C]-sucrose to orphan shoot (twigs) and [^14^C]-chasing revealed substantial radiolabel incorporation in tropinone and tropine, confirming the *de novo* synthesizing ability of the aerial tissues. This inherent independent ability heralds a conceptual novelty in the backdrop of classical view that these tissues acquire the alkaloids through transportation from roots rather than synthesis. The TR-I gene expression was found to be up-regulated on exposure to signal molecules (methyl jasmonate and salicylic acid) and on mechanical injury. The enzyme's catalytic and structural properties as well as gene expression profiles are discussed with respect to their physiological overtones.

## Introduction

*Withania somnifera* (popularly known as Ashwagandha or Indian ginseng), is one of the most reputed medicinal plants of the Indian systems of medicine and forms essential constituent of >100 herbal and nutraceutical formulations [1]. The herb possesses pharmacological activities like anti-arthritic, cognitive function improvement in geriatric states and recovery from neurodegenerative disorders [2], [3]. Phytochemically, the plant is unique in producing several types of secondary metabolites such as withanolides, withanamides, and alkaloids [2], [3], [4], [5], [6], [7], [8], [9], [10]. Plant alkaloids based on different chemical skeletons like tropane, indole, benzoisoquinoline [11], [12], [13], [14] have been occupying a unique significance in human activities from cultural and spiritual to therapeutics. In fact, use of the word ‘alkaloids’ had conventionally become so ingrained to describe a herbal febrifuge that even today, herbs are being traded in non-standardized market in terms of their ‘alkaloid content’ while actual characteristic metabolite of the herb could be non-alkaloidal in its chemical nature.

Tropane alkaloids represent some of the earliest known and used alkaloidal drugs isolated from a range of Solanaceae medicinal plants like *Atropa, Hyoscyamus*, *Duboisia*, and *Datura* [15], [16]. Their pre-eminence also led to initiation of phytochemical investigation of *W. somnifera*, the most noted medicinal plant of Solanaceae, for alkaloids. Consequently, tropane alkaloids like tropine, pseudotropine, 3-tropyl-tigloate were the first set of phytochemicals reported from the plant [2]. Even bioactivities of some of them probably constituted earliest such reports on phytochemical-specific studies on the plant in 1950s [17]. However, since then, occurrence of this group of compounds was neither ever validated nor did the list of tropane alkaloids expand. Instead, withanolides, the C_28_ compounds of triterpene metabolic ancestry were reported and a prolific series of investigations described their diverse pharmacological activities [2], [5], [18], [19], [20], [21], [22], [23]. This also prompted the initiation of metabolic pathway and functional genomics studies on the plant during last decade [7], [24], [25], [26], [27], [28], [29]. Nevertheless, the usual comparatively lower pharmacological efficacies of pure withanolide(s) than the extracts may also imply some complementary or enhancive effect of other phytochemicals possessed by the plant, implying importance of the discipline of identifying and understanding components of the ‘systems phytochemical biology’ in the present ‘ómic era of biology’ [2]. This together with the reports on identification of metabolites related to tropanes and pseudotropanes (including calystegines, the bioactive signature compounds of several food members of Solanaceae family) in the plant, accentuates importance of investigation of biosynthesis of these overlooked phytochemicals in the herb [2], [30], [31], [32].

**Figure 1:**
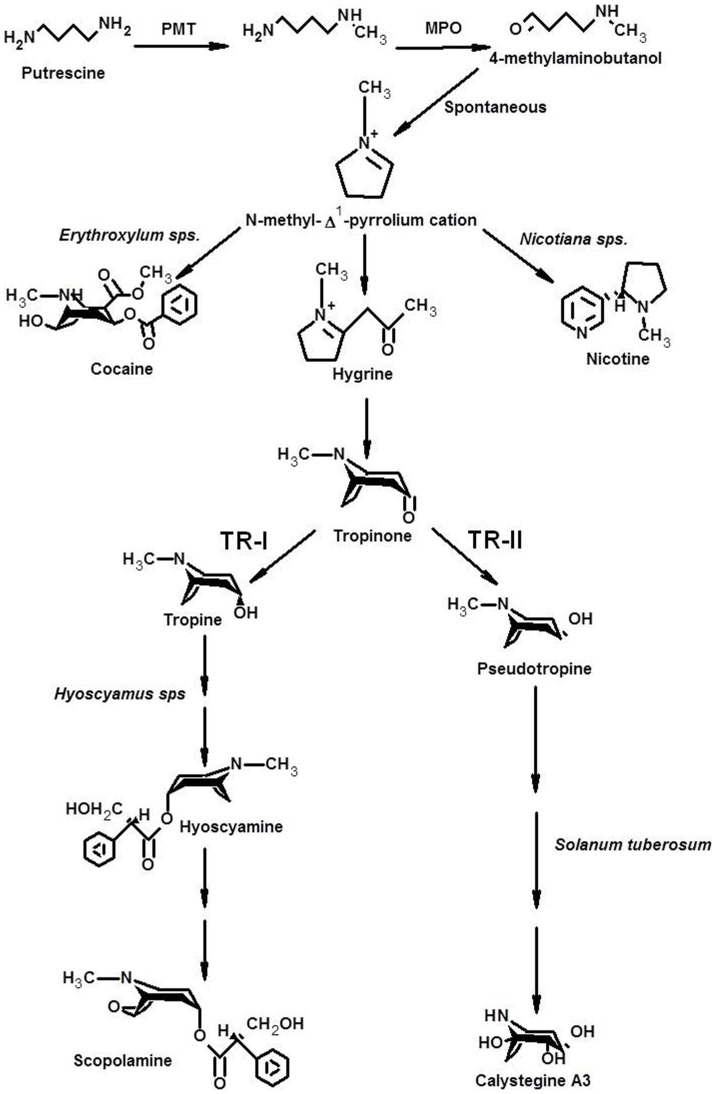
Schematic representation of metabolic pathway of tropane alkaloid biosynthesis. TR-I, Tropinone reductase I; TR-II, Tropinone reductase II.

Tropinone reductases (TRs) are NADPH-dependant enzymes belonging to protein family of SDRs (short-chain dehydrogenases/reductases) [33]. TRs catalyse reduction of tropinone into tropine and thereby enabling biosynthesis of tropane and pseudotropane alkaloids (Figure 1). Two classes of TRs are reported that catalyzes stereo-specific conversion of tropinone into tropine (TR-I) and pseudotropine (TR-II) [34], [35]. Both, TR-I and TR-II, are present together in any given tropane alkaloid-producing species and varying expression levels of the two TRs in the tropane alkaloid-producing species, may govern the precursor metabolite flux distribution at the branch point across the two metabolic streams [34], since no inter-conversion between tropine and pseudotropine has been observed *in vivo* [36].

In view of recent immense global interest in *W. somnifera*, a lot of biochemical and functional genomics knowledge has become available with respect to biosynthesis of its withanolides [7], [24], [25], [26], [27], [28], [29]. Whilst tropane alkaloids of *W. somnifera*, despite being considered its important secondary metabolites, have not been studied for the alkaloid biosynthesis. Biosynthetic studies on other tropane alkaloid producer Solanaceae plants like *Hyoscyamus muticus*, *Atropa belledona*, *Hysocyamus niger* have led to the concept of roots-limited synthesis of these compounds followed by their translocation (acropetal transport) through xylem to leaves for sequestration [37]. This prompted us to initiate investigation of the tropane alkaloid biosynthesis in *W. somnifera* through classical molecular biology approach. Here, we report biochemical, functional, genomic and physiological aspects of a tropinone reductase involved in tropane alkaloid biosynthesis in aerial tissues of the plant. The investigation comprises heterologous expression of the tropinone reductase gene cloned from *W. Somnifera* and discerning tissue-wide profiles of gene-expression, as well as catalytic kinetics of the recombinant tropinone reductase catalyzing NADPH-dependent conversion of tropinone into tropine. The study revealed the conceptual novelty of inherently independent metabolic competence of aerial tissues of the plant to synthesize tropane alkaloids. The inference was confirmed by *in vivo* feeding of radio-labelled U-[^14^C]-sucrose to orphan shoots (twigs) of the plant and tracing the label incorporation in tropinone and tropine. A comparative three-dimensional structural model of the catalytic protein and active-site residues landscape has also been developed for the enzyme. To our knowledge, this pertains to be the first report on alkaloid pathway genes and enzymes from *W. somnifera* and entails novel aspects of tropane alkaloid biosynthesis *in planta*.

## Materials and Methods

### Plant Material

*Withania somnifera* Dunal cv. NMITLI-118 plants were raised at experimental farm of Central Institute of Medicinal and Aromatic Plants, Lucknow (India) following standard agronomic practices and sampled for gene cloning, expression analysis and metabolite assays. For RNA isolation young roots of 2–5 mm thickness were used. For transcript abundance and metabolite analysis, roots and shoots of 2–5 mm thickness, young leaves (4–5 mm expansion), mid expanded leaves (10–15 mm), fully expanded leaves, flowers and berries (immature, mature and ripened) were harvested concurrently.

### RNA Isolation and cDNA Synthesis

The tissue was immersed in liquid Nitrogen immediately after sampling, and total RNA was isolated using TRI reagent (Sigma-Aldrich, USA) following the manufacturer's protocol. First strand cDNA was synthesised using RevertAid™ cDNA synthesis kit (Fermentas Life Sciences, USA) as per the manufacturer's instructions. Root cDNA library was synthesized using SMARTer^TM^ PCR cDNA synthesis kit (Clontech Laboratories Inc, USA) for the isolation of 5′ region.

### Cloning of Tropinone Reductase (WsTR-I) Gene

Several degenerate primer pairs, designed from the conserved regions, identified by alignment of the known nucleotide sequences of tropinone reductase-I (TR-I) genes, were used for PCR amplification to score putative *W. somnifera* TR-I (*WsTR-I*). RT-PCR with primer pair TRIF2 (5′-TTC CTR AAT AAT GCM GGD GTG 3′) and TRd1CterR (5′-TYA AAA YCC ACC ATT AGC TGT RAA TCC- 3′) resulted in amplification of a DNA fragment that was cloned in pJET1.2/blunt vector using ClonJET PCR cloning kit (Fermentas Life Sciences, USA) and sequenced. This sequence was used to carry out 3′ RACE (primer pair of TRd1CterF: 5′-AAT GGG CCA ARG ASA AYA TTC GGG TC-3′ and 3′ AP: 5′ -GGC CAC GCG TCG ACT AGT AC (T)_17_- 3′). The 3′ RACE PCR product (368 bp) was cloned and sequenced. 5′ RACE was performed with SMARTer cDNA using gene specific primers and SMARTer™ II A oligonucleotide (supplied with kit) following the manufacturer's manual. First PCR was performed with TRIUR1 (5′- TAA TTG CTG CTT CAA CGA GAG GG -3′) and CDS II A primers. Three rounds of nested PCR amplification were carried out using gene specific reverse primers TRIUR2 (5′- ACT CCT GGA GCA ACT GAA TTG ACC -3′), TRIUR3 (5′-AAG GCA GTG CTG AAA ATC CAG C-3′) and TRIUR4 (5′-ACA TTG CCA TTT TGA GAA GCC TTC-3′) with CDS II A (provided in the kit) as universal forward primer. Amplified fragments were cloned and sequenced.

The sequences of internal partial fragment and 3′ RACE and 5′ RACE PCR products were assembled to obtain full-length coding (ORF) sequence of *WsTR-I*. Full length cDNA was obtained by performing a PCR using a pair of gene specific primers extended at 5′ for incorporation of restriction sites for *Nde*I (5′ primer: 5′-*CAT ATG* GAT GAA TCA AGC GTT TGC ATG-3′) and *Bam*HI (3′ primer: 5′-*GGA TCC* TTA AAA TCC ACC ATT AGC TG-3′). The PCR product was cloned into pJET1.2/blunt vector and sequenced for confirmation.

### Computational and Phylogenetic Analysis

The BLAST tool of NCBI (National Center for Biotechnology Information; http://blast.ncbi.nlm.nih.gov/Blast/) was used to find similarity for *WsTR-I* cDNA with other TR sequences in the database. ORF Finder graphical analysis tool (www.ncbi.nlm.nih.gov/projects/gorf) was used to predict coding sequences. ClustalW2 tool (http://www.ebi.ac.uk/Tools/msa/clustalw2) was used for sequence alignment. A phylogenetic tree was constructed by the maximum likelihood method and subjected to bootstrap analysis using MEGA5 software (http://www.megasoftware.net).

A three-dimensional structure of WsTR-I protein, derived from its amino acid sequence, was developed at SWISS-MODEL WORKSPACE in automated mode. DsTR-I (1ae1.pdb) was used as model template. The tertiary structure produced by SWISS-MODEL was superimposed onto the modelled structure of DsTR-I (1ae1.pdb) and DsTR-II (1ipf.pdb) using UCSF Chimera package. To transfer tropinone to the developed model, another model was developed by aligning WsTR-I model with DsTR-I (1ae1.pdb) and DsTR-II (1ipf.pdb) following energy minimization. The model was explored by Ligand Explorer 4.0 to examine the active sites. For comparison of the active site of WsTR-I in the generated model with DsTR-I and DsTR-II, all the three models were aligned and their active sites were analyzed in Pymol.

### *WsTR-I* Transcript Abundance Analysis

Gene specific primers were designed with Primer Express software version 2.0 (Applied Biosystems, USA) and custom synthesized. First strand cDNA was synthesized from 2 µg total RNA using Revertaid^TM^ cDNA synthesis kit (Fermentas, USA) and oligo dT primers. The reaction was set up with ∼50 ng cDNA, 5 pmole of each forward and reverse gene specific primers, 10 µl of Power SYBR Green PCR master mix 2X (Applied Biosystems, USA) and the final volume was adjusted to 20 µl. The reactions were set in triplicates. Specificity of the reaction was verified by melting curve analysis. The relative mRNA levels for the genes were quantified taking β-actin as endogenous control. The thermal cycling parameters were: initial denaturation (95°C for 10 min); and 40 amplification cycles (95°C for 15 s; and 60°C for 1 min) followed by 95°C for 15 s; and 60°C for 1 min to study the dissociation curve of amplified products. The reactions were carried out on StepOne^TM^ Real Time PCR System with 48-well block module (Applied Biosystems, USA). Relative quantification of gene transcript abundance was done following the comparative ΔΔ C_T_ method (where relative quantity or RQ = 2^−ΔΔ CT^).

### Gene Expression Modulation by Signal Molecules and Mechanical Injury

To analyze modulation of expression of *WsTR-I* gene on exposure to methyl jasmonate (MeJa) and salicylic acid (SA), *W. somnifera* leaves were placed onto a blotting paper *a priori* soaked in either methyl jasmonate (MeJa, 50 µM) or salicylic acid (SA, 1 mM). For control, only sterile and deionized (Milli Q) water was used for soaking. For discerning the impact of wounding (mechanical injury) on the gene expression, leaves were mechanically injured with sterile scalpel and placed onto blotting paper soaked in sterile and deionized (Milli Q) water. Samples were collected after 1 and 6 h, frozen in liquid N_2_, subjected to RNA isolation followed by cDNA synthesis and quantification of transcript abundance by qRT-PCR as described above.

### Heterologous Expression of WsTR-I in *E. coli*

The cloned *WsTR-I* cDNA fragment was sub-cloned into linearized pET28a protein expression vector at *Nde*I and *Bam*HI site. Competent cells of *E. coli* BL21 (DE3) (Novagen, USA) were transformed with *WsTRI*-pET28a construct. Over-expression of *WsTR-I* was achieved by induction with 1 mM IPTG for 18 hours at 30°C. The cells were subjected to protein extraction followed by SDS-PAGE analysis to identify the induction-over expressed polypeptide translated by *WsTR-I*.

### Purification of the Recombinant Enzyme

To isolate the *WsTR-I* recombinant enzyme in large amount, 1 L culture of recombinant *E. coli* BL21 (DE3) cells were induced by the addition of 1 mM IPTG. Soluble fraction was collected as supernatant of the lysate centrifugation (20000×g for 1 h). The His-tagged recombinant protein was affinity purified and eluted fractions were screened for protein (A_280_) as well as catalytic activity. Fractions containing tropinone reductase activity were pooled. Further, traces of contaminating proteins in the recombinant enzyme preparation were removed by gel filtration chromatography through Sephadex G-75 (GE Health Sciences, USA) using 0.1 M buffer of MES-NaOH buffer (pH 6.7) containing 0.1 M NaCl, as equilibration and elution buffer. Eluted fractions (1.0 ml each) were screened for protein (A_280_) and the enzyme activity and catalytically active fractions were pooled. The enzyme preparation was analyzed for protein concentration and SDS-PAGE polypeptide profile.

### Native Catalytic Protein Molecular Weight Determination

The native molecular mass of the catalytically active tropinone reductase-I was determined by size exclusion column chromatography through a calibrated column (70.0 cm×0.6 cm) packed with Sephadex G-150 (GE Health Sciences, USA) and pre-equilibrated with 0.1 M MES-NaOH buffer (pH 6.7) supplemented with 0.1 M NaCl at a flow rate of 5 ml/h. The column was calibrated using ovalbumin (43 kDa), bovine serum albumin (BSA) (66 kDa) and alcohol dehydrogenase (150 kDa) as size markers. Blue Dextran (2000 kDa) was used to determine void volume (V_o_) of the column. Elution volume for the catalytically active enzyme was determined from the catalytic activity profile in the eluted fractions on chromatography of the enzyme preparation. K_d_ values for the marker and test proteins were determined from the elution volume (V_e_) and column parameter values (V_0_, V_t_). Molecular mass of the enzyme was computed from the calibration plot of log molecular weight *versus* K_d_.

### Validation of Catalytic Reaction Product

To establish the identity of the product of the enzyme catalyzed reaction, activity assays were carried out in large volume. A reaction volume of 10 ml was set with the composition of 200 mM tropinone, 200 µM NADPH and 50 mM MES-NaOH buffer. Recombinant WsTR-I protein (5 µg) was added to the mix and incubated at 30°C for 30 min. A control reaction was set up excluding WsTR-I protein. Tropanes were extracted from the assay mixture with equal volume of chloroform (twice). Chloroform extracts were evaporated to dryness and residues were dissolved in 1.0 ml of HPLC grade methanol and were analyzed by Gas chromatography (GC). GC was performed with PerkinElmer Claurus 500 GC-System equipped with Elite-5 (5% diphenyl) dimethylpolysiloxane capillary column (50 m, 0.32 mm i.d., 1.00 µm film thickness). The injection volume was 1 µl with a split ratio of 20∶1. Nitrogen was used as the carrier gas with a constant flow of 1 ml min^−1^. The inlet temperature was 250°C. For detection, a flame ionisation detector was used at a temperature of 310°C. GC was run on the program: initial temperature 65°C, 10°C min^−1^ up to 150°C, 2 min isotherm, 10°C min^−1^ up to 240°C, post run at 240°C for 5 min.

To authenticate the GC results, reaction product was analyzed by GC-MS. Gas chromatography was performed with PerkinElmer Claurus 680 system equipped with DB-5 column (30 m, 0.25 mm i.d., 0.25 µm film thickness). The injection volume was 1 µl with a split ratio of 100∶1. Helium was used as the carrier gas with a constant flow of 1 ml min^−1^. The injector temperature was 250°C. The mass spectrometer was a Claurus SQ 8C (Perkin-Elmer) with the El mass range set at 40–450 AMU.

#### Sodium borohydride reduction of tropinone to tropine and pseudotropine

25 mg of tropinone was dissolved in 15 ml of methanol. 25 mg of NaBH4 was added into the solution while stirring the mixture continuously on ice-bath. Progress of the reaction was checked by TLC. For TLC, 5 µl of the reaction mixture was loaded onto a pre-coated silica gel 60 TLC plate (Merck). TLC was performed in mobile phase of chloroform: methanol: ammonia (30∶5∶1) and developed with a modified Dragondorff' reagent [38]. Tropinone and tropine standards (Sigma-Aldrich, USA) were run along with samples for identification. Once the reaction was complete, methanol was vacuum evaporated. Residues were dissolved in 10 ml of ethyl acetate and partitioned twice with equal volume of water. Ethyl acetate fraction was vacuum evaporated, dissolved in 1 ml of methanol and subjected to GC analysis as discussed previously. Validation of GC results was done by GC-MS. Gas chromatography was executed with PerkinElmer Claurus 680 system equipped with Elite 5MS (30 m×0.25 mm×0.25 µm). The injection volume was 1 µl with a split ratio of 100∶1. Helium was used as the carrier gas with a constant flow of 1 ml min−1. The injector temperature was 250°C. The mass spectrometer was a Claurus SQ 8C (Perkin-Elmer) with the El mass range set at 40–450 AMU.

### Enzyme Assay

Tropine forming tropinone reductase (TR-I) activity (forward reaction) was assayed spectrophotometrically (A_340_) by following the rate of tropinone-dependent oxidation of NADPH at 30°C. The standard assay mixture (1.0 ml) contained 2.5 mM tropinone, 100 µM NADPH and 100 mM MES-NaOH buffer (pH 6.7). The reaction was started by the addition of tropinone. Control was run in parallel wherein tropinone was omitted from the reaction mixture and used for the corrections in the experimental observation. In case of reverse reaction, tropinone and NADPH were replaced by tropine (10 mM) and NADP^+^ (0.3 mM), respectively. The activity was monitored spectrophotometrically as the rate of tropine-dependent reduction of NADP^+^ by following the increase in A_340_.

The enzyme activity was expressed as international units (IU), *i.e*., micromoles of product formed per min under the assay conditions, considering the absorption coefficient of NADPH at 340 nm. For analysis of catalytic kinetics of the enzyme, the standard assay mixture composition and conditions were altered as required for the specific kinetic and biochemical characterization of the enzyme. All the assays were set in triplicates and mean values with standard deviation (± SD) were used in kinetic analyses.

### Metabolite Analysis

Fresh tissues (20 g) were harvested from the field and powdered in liquid N_2._ The fine powder was soaked in 100 ml of 100% methanol overnight and filtered. Procedure was repeated thrice. Methanol extracts (filtrates) were pooled and partitioned thrice with equal volume of hexane. Methanol fractions were evaporated to the dryness. Dried extracts were dissolved in methanol (Merck) and analyzed by TLC as well GC. Quantification was done following single point external standard method in GC.

### *In Vivo* incorporation of U-[^14^C]-Sucrose to Orphan Shoots and Radiolabel Tracing in Tropanes

To assess the metabolic competence of aerial tissues of *W. somnifera* to independently synthesize tropine, orphan shoots (twigs) of the plant were vascularly fed with U-[^14^C]-sucrose (obtained from BRIT, Board of Radiation and Isotope Technology, India). A total of 0.3 MBq radioactivity was fed. After the chase period of 72 h, leaves were separated from stem and tropanes were extracted as detailed above. Concentrated methanolic extracts were loaded on to a preparative silica gel TLC plate (Merck Chemicals) and ran as described earlier. Labelled metabolites was localized on TLC by scanning on a phosphoimager (Typhoon Tri+ Variable mode imager, GE Healthcare Lifesciences) and matched with co-run iodine stained standards (tropine and tropinone). Additionally, radiolabel incorporation into the tropanes was quantitated by scrapping the silica-gel of samples lanes corresponding to the position of standards, extracting with methanol and assaying level of radioactivity in a liquid scintillation counter (MicroBeta 2 2450; PerkinElmer).

### Statistics and Other Analyses

The analyses were made in triplicate and are presented as mean values with standard deviation (± SD). Protein concentration was determined by the method of Lowry [39] using BSA as standard.

## Results and Discussion

### Cloning of WsTR-I and Sequence Analysis

A cDNA library of *W. somnifera* roots was used as template for a degenerate primed PCR amplification of *TR-I*. It resulted in a 471 bp fragment with highly significant sequence homology with *D. stramonium TR-I*. 3′ RACE of the fragment resulted in a 368 bp amplicon that shared nucleotide sequence homology with 3′ region of *D. stramonium TR-I* sequence. 5′ RACE with gene specific primers using a SMARTer^TM^ cDNA library gave a 569 bp PCR product having sequence similarity with 5′ region sequence of *TR-I* of *Anisodus acutangulus*. Collation of sequences of the three PCR products resulted in an assembly of 1050 bp cDNA (*WsTR-I*), spanning the gene transcription start and stop codons. Gene specific primers flanked with restriction sites were designed at the start and stop site of the assembled *WsTR-I* sequence. PCR with these primers resulted in an 822 bp cDNA fragment containing complete ORF, that showed maximal homology with *TRI* sequences available in the public domain database. The ORF represented a polypeptide of 273 amino acids with a calculated molecular mass of 29.41 kDa and theoretical isoelectric point (pI) of 5.99. The full length cDNA sequence of the gene was deposited with NCBI database (GeneBank Accession No. KF360055).

**Figure 2:**
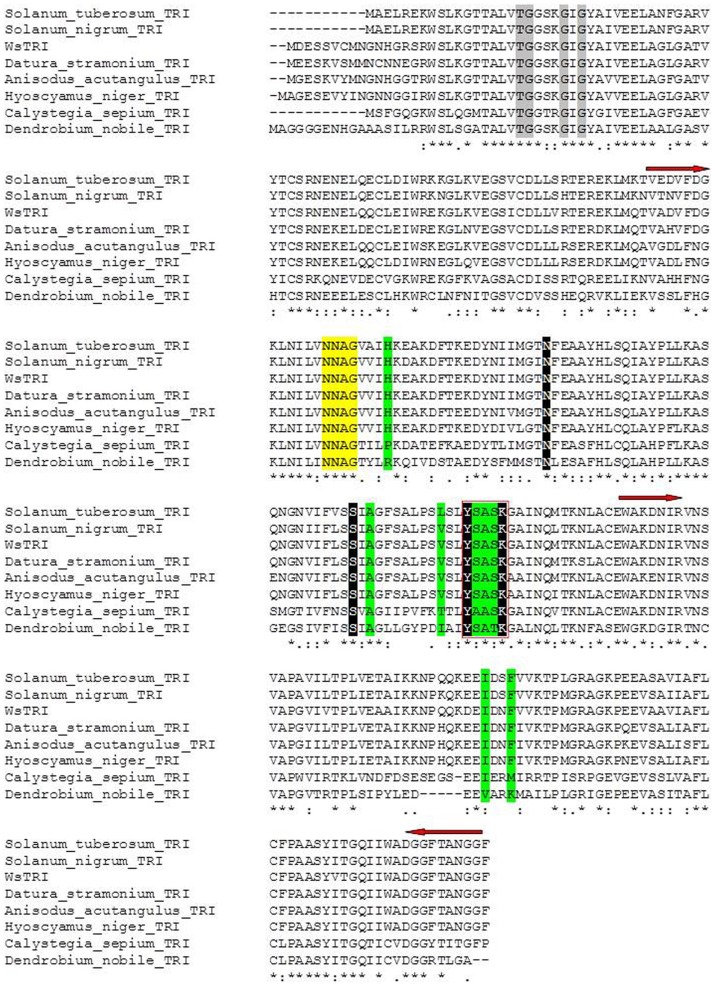
Comparison of tropinone reductase from *W. somnifera* (WsTR-I) with known tropinone reductases by multiple sequence alignment. Asterisks indicate identical amino acid positions. Arrows denote the regions selected to design degenerate primers. Residues highlighted in grey are involved in co-factor binding. Box designates the signature YXXXK motif of short chain dehydrogenases/reductases (SDRs) and sequence highlighted in black denotes catalytic tetrad. Green highlighted residues participate in tropinone binding. Yellow highlight indicates the residues conserved in SDRs.

BLAST (http://www.ncbi.nlm.nih.gov/BLAST) results of the deduced amino acid sequence of *WsTR-I* revealed its high homology (Figure 2) with the amino acid sequences of *TR*s from other plants; for example, 90%, 89%, 87% and 86% similarity with *StTR-I* (*Solanum tuberosum* TR-I; accession number CAC34420), *DsTR-I* (*D. stramonium* TR-I; accession number AAA33281), *AaTR-I* (*A. acutangulus* TR-I, accession number ACB71202), and *HnTR-I* (*Hyoscyamus niger* TR-I; accession number BAA13547), respectively. It exhibited relatively lower sequence homology with pseudotropine forming tropinone reductase (called TR-II). The values of sequence similarity with TR-II were 84%, 83%, 82% and 81% with *StTR-II* (*S. tuberosum* TR-II; accession number CAC19810), *AaTR-II* (*A. acutangulus* TR-II, accession number ACB71203), *HnTR-II* (*H. niger* TR-II, accession number P50164) and *DsTR-II* (*D. stramonium* TR-II, accession number AAA33282), respectively. This level of sequence similarity among the TRs indicates, *per se*, high level of sequence conservation, as reported earlier [36], [38], [40], [41], [42], [43]. As expected, the amino acid sequence of *WsTR-I* also exhibited significant similarity with a specific group of dehydrogenases/reductases from non-Solanaceous plants such as *Arabidopsis*, *Vitis vinifera*, *Populus trichocarpa* that are known so far, to be non-producers of tropane alkaloids. These genes/enzymes are called short-chain dehydrogenases/reductases (SDRs) and are identified by some characteristic sequence motifs. They form a well-established enzyme family of oxidoreductases but distinct from the classical functionally similar oxidoreductase enzyme families and sub-families [33]. The characteristic motifs of the SDRs were also evident in *WsTR-I* sequence, for example, NADPH binding TGXXXGXG motif [33]. This motif was present at 27–34 position in *WsTR-I*. Similarly, NNAG motif of SDRs was located at position 105–108 in *Withania* TR-I protein (Figure 2). The tyrosine residue of catalytic sequence motif YXXXK of SDRs [33] was observed at position 171 of WsTR-I (Figure 2). The tyrosine residue of the later motif is considered essential for the catalytic activity of TRs, as discerned from the crystal structures and site-directed mutagenesis studies [35], [41], [45]. The substrate orientation in the active site is crucial for the stereo-specific reduction of tropinone (27). Site-directed substitution experiments in *D. stramonium* and *H. niger* had revealed involvement of five residues, His^112^, Ala^160^, Val^168^, Ile^223^ and Phe^226^, in stereo-specificity of the respective TR-Is [40]. These residues were observed to be conserved at analogous positions in WsTR-I as well. Thus, the cloned WsTR-I may represent a functional form of TR-I of *W. somnifera* catalyzing reduction of tropinone to tropine through similar catalytic stereo-specificity of substrate orientation and product generated as in case of other TR-Is.

### Heterologous Expression and Purification of Recombinant Enzyme

**Figure 3:**
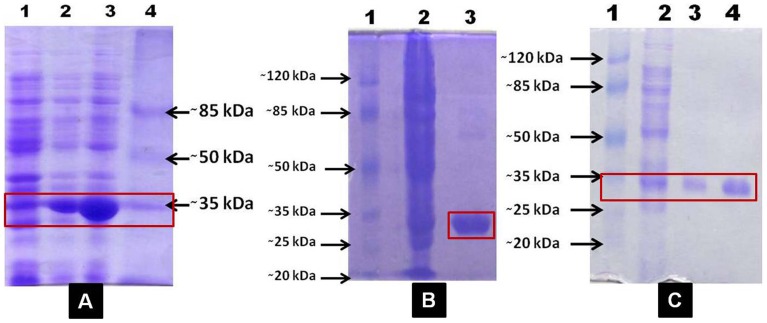
SDS-PAGE analysis of His-tagged recombinant tropine forming tropinone reductase of *W. somnifera* (WsTR-I). A, induced expression of WsTR-I in *E. coli*: lane 1, un-induced culture extract; lane 2, induced soluble fraction; lane 3, induced insoluble fraction; lane 4, protein molecular weight marker; B, lane 1, enzyme eluted from affinity Column (Ni^++^-NTA); lane 2, crude soluble fraction; lane 3, protein molecular weight marker; C, lane 1, protein molecular weight marker; lane 2, crude soluble fraction; lane 3 and 4, enzyme eluted from gel filtration column.

The full length *WsTR-I* cDNA was over-expressed in *E. coli* BL21 (DE3) cells harbouring pET28a as the protein expression vector. Recombinant His_6_-WsTR-I was homogeneity purified by Ni^++^-NTA affinity chromatography followed by size exclusion chromatography (Figure 3). Purity of the enzyme was ascertained by SDS-PAGE, wherein a 29 kDa polypeptide was detected as sole constituent of the protein preparation (Figure 3). The purified WsTR-I was biochemically characterized for its catalytic and kinetic properties.

### Biochemical Characterization

#### Catalytic activity and reaction product identification

**Figure 4:**
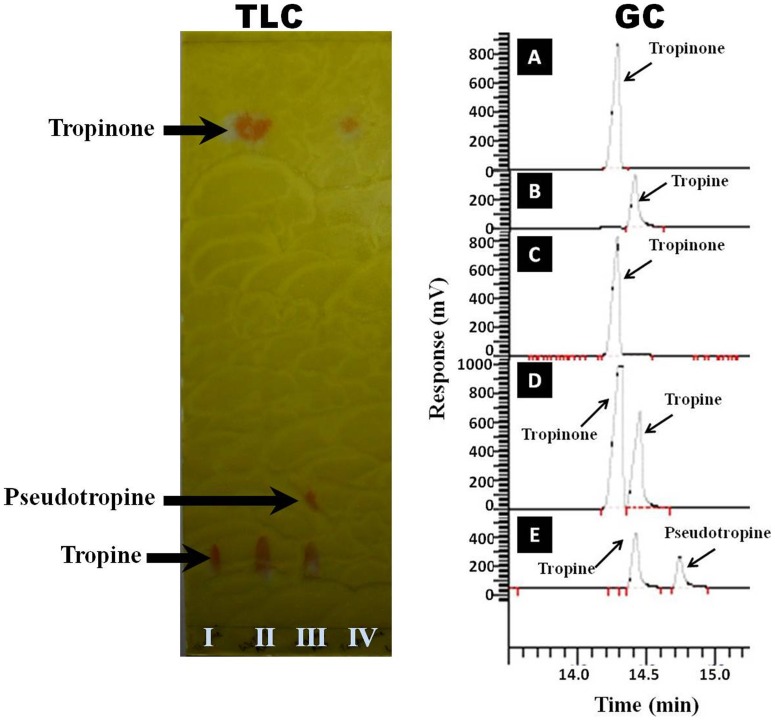
Identification of the reaction product of recombinant WsTR-I with tropinone as substrate. TLC: I, tropine standard; II, WsTR-I reaction mixture after catalytic reaction termination; III, NaBH_4_ reduction products (tropine and pseudotropine) of tropinone; IV, tropinone standard. GC: A, authentic tropinone; B, authentic tropine; C, control (enzyme minus) assay mixture of WsTR-I; D, experimental (complete) assay mixture of WsTR-I assay; E, NaBH_4_ aided reduction products (tropine and pseudotropine) of tropinone.

**Figure 5:**
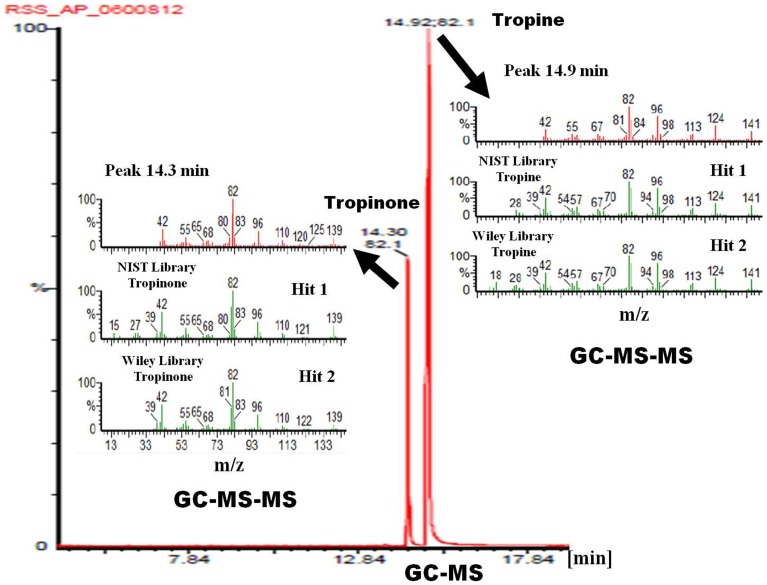
Gas chromatography-mass spectrometry analysis (GC-MS) of the enzymatic reaction mixture with tropinone as substrate. A, MS-MS spectra of first GC peak and their matching with NIST and Wiley library leading to identification of the sample (enzymatic reaction mixture) peak (R_t_14.3 min) as tropinone (substrate of the enzymatic reaction) and peak (R_t_14.9 min) as tropine (product of the enzymatic reaction). B, MS-MS spectra of the second GC peak and their matching with NIST and Wiley library.

Spectrophotometric (tropinone-dependent oxidation of NADPH into NADP^+^) assays clearly exhibited the association of tropinone reductase activity with the WsTR-I protein. TLC and GC-based identification of tropine as product in the enzymatic reaction mixture (Figure 4) established stereo-specificity of the reaction catalyzed by enzyme as the tropine forming tropinone reductase i.e. TR-I. To further substantiate authenticity of the WsTR-I catalysis, the product of enzyme catalyzed reaction was identified by GC-MS analysis. Congruent to the GC-based separation, the alkaloidal constituents of the reaction mixture at the end of the enzyme assay were identified as tropinone (MS *m/z* 139, 125, 120, 110, 96, 83, 82, 80, 68, 65, 55, 42) and tropine (MS *m/z* 141, 124, 113, 98, 96, 84, 82, 81, 67, 55, 42), based on full matching of the observed mass fragment patterns (MS-MS data) with the respective molecules in the standard compound in NIST as well as Wiley library (Figure 5). Furthermore, in parallel, it was established that product of the reaction catalyzed by WsTR-I was solely tropine, and its isomer, pseudotropine (product of TR-II type catalysis) was not generated by the enzymatic reaction. For this, the WsTR-I reaction product was compared with a mixture of tropine and pseudotropine, that was generated by NaBH_4_ aided chemical reduction of tropinone. NaBH_4_ mediated reduction of tropinone yielded two Dragendorff reagent positive spots on TLC of the reaction products (Figure 4), as expected. One of these spots (lower R_f_) corresponded to tropine standard, while another (higher R_f_) was tentatively assigned to be pseudotropine. Similarly, gas chromatographic (GC) analysis of the chemical reaction products also resulted into two well resolved peaks with retention times of 14.41 and 14.74 min, respectively (Figure 4). One of these peaks was corroborated to be tropine, based on its same retention (14.41 min) as that of authentic tropine sample analyzed under identical conditions. Further, on GC-MS-MS analysis, both peaks were found to be of same mass (141) and displayed identical mass spectra that matched to authentic tropine (Figure 5). Therefore, they were designated as tropine and pseudotropine, respectively. Drager et al. [46] have also reported that tropine and pseudotropine display clearly different GC retention times but identical mass spectra. Accordingly, the second product of the chemical reduction reaction was confirmed as pseudotropine (with GC retention time of 14.74 min), well resolved from tropine (Figure 4 and Figure 5). Thus, TLC, GC and mass spectrometric data on the chemical (NaBH_4_) and enzymatic reduction products of tropinone (Figure 4 and Figure 5) fully ascertained that tropine was the sole product of the WsTR-I enzyme catalyzed biochemical reaction. The recombinant WsTR-I was characterized further for its catalytic and structural characteristics.

#### Optimum pH and thermostability

**Figure 6:**
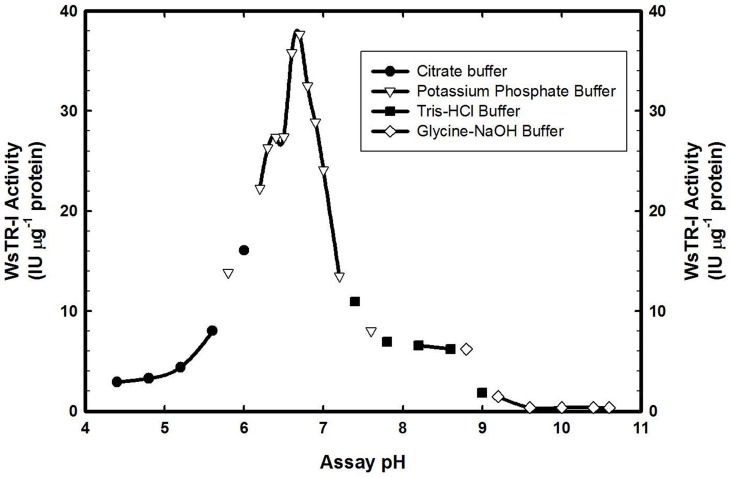
Effect of pH on the catalytic activity of tropine forming tropinone reductase (WsTR-1) of *W. somnifera*.

WsTR-I had pH optima of 6.7 with a relatively narrow pH range of significant catalytic activity (Figure 6). Though, such a narrow pH range for TR-I activity has been previously reported for HnTR-I, StTR-I and DsTR-I [35], [41], [45], but their pH values of optima activity are reported to be 5.9, 6.0, and 7.0, respectively (Table 1). WsTR-I was meta stable with respect to its thermal inactivation and retained about 75% of its catalytic activity up to 50°C (Figure S1A). But, the catalytic activity was rapidly and substantially lost at higher temperatures with barely 4.27% activity remaining on incubation at 60°C. Nevertheless, presence of glycerol (20%) and sucrose (20%) considerably improved the enzyme thermostability whilst presence of 2-mercaptoethanol did not display any profound effect (Figure S1B).

**Table 1:** Comparative account of catalytic parameters (pH optima and K_m_) of *W. somnifera* tropine forming tropinone reductase (WsTR-I) with other plant TRs reported from plants.

Enzyme	pH Optima	K_m_ (mM)	Reference
WsTR-I*	5.7	1.27	Present study
StTR-I*	6.0	0.188	Kaiser et al. [46]
StTR-II*	6.4	0.033	Keiner et al. [57]
DsTR-I*	7.0	0.117	Nakajima et al. [41]
DsTR-II*	7.0	0.048	Nakajima et al. [41]
DsTR-I#	6.4	1.3	Portsteffen et al. [58]
DsTR-II#	6.4	0.11	Portsteffen et al. [58]
HnTR-I*	5.9	1.01	Hashimoto et al. [35]
HnTR-II*	5.9	0.034	Hashimoto et al. [35]
HnTR-II#	5.8	0.035	Drager et al. [46]
CoTR-I*	8.0	3.29 (at pH 6.4)	Brock et al. [48]

* Recombinant protein expressed in *E. coli*.

# Native protein purified from the plant source.

#### Substrate saturation kinetics

WsTR-I displayed a hyperbolic substrate saturation curve for the substrate and cofactor, for both forward as well as reverse reaction (Figure S2 and Figure S3). It signifies that the enzyme followed normal Michaelis-Menten kinetics of catalysis. The double reciprocal plots of the enzyme for the forward and reverse reaction substrates (tropinone and tropine, respectively) as well as cofactors (NADPH and NADP^+^) gave an estimate of their K_m_ values as: tropinone (1.27 mM), tropine (114.9 mM), NADPH (0.43 µM) and NADP^+^ (96.15 µM ) (Table 2). This profile of Michaelis-Menten constants suggested that the catalytic reaction was predominantly favoured in forward direction (tropine formation). Further, the enzyme had higher affinity for cofactors than for its substrates. This is physiologically understandable as the tissue concentration of cofactors (NADPH and NADP^+^) cannot be operated at high levels as they are some of the metabolically most dynamic metabolites involved in enormous reactions of primary and secondary metabolism. Further, nor the levels of these cofactors can vary widely under normal physiological conditions as their ratio (NADPH/NADP^+^) governs the overall redox status of the tissue and forms an important index of developmental and metabolic homeostasis [47]. Thus, the metabolic step of tropine formation may be regulated by, besides through the transcript and protein levels, the tissue concentration of tropinone.

**Table 2:** Kinetic parameters of *Withania somnifera* tropine forming tropinone reductase (WsTR-I) catalyzed reaction.

Substrate	K_m_	K_cat_ (Monomer, single active site)	K_cat_/K_m_(M^−1^.S^−1^)	K_cat_(Dimer, single active site)
Tropinone	1.27 mM	9.51	7.49×10^3^	19.03
NADPH	0.43 µM	33.34	7.74×10^7^	66.68
*N-*methyl-piperidine	1.74 mM	8.97	5.17×10^3^	17.95
3-methyl-cyclohexanone	2.11 mM	9.23	4.38×10^3^	18.46
Tropine	114.94 mM	1.67	0.14×10^2^	3.33
NADP^+^	96.15 µM	0.54	5.59×10^2^	1.06

#### Substrate specificity

**Figure 7:**
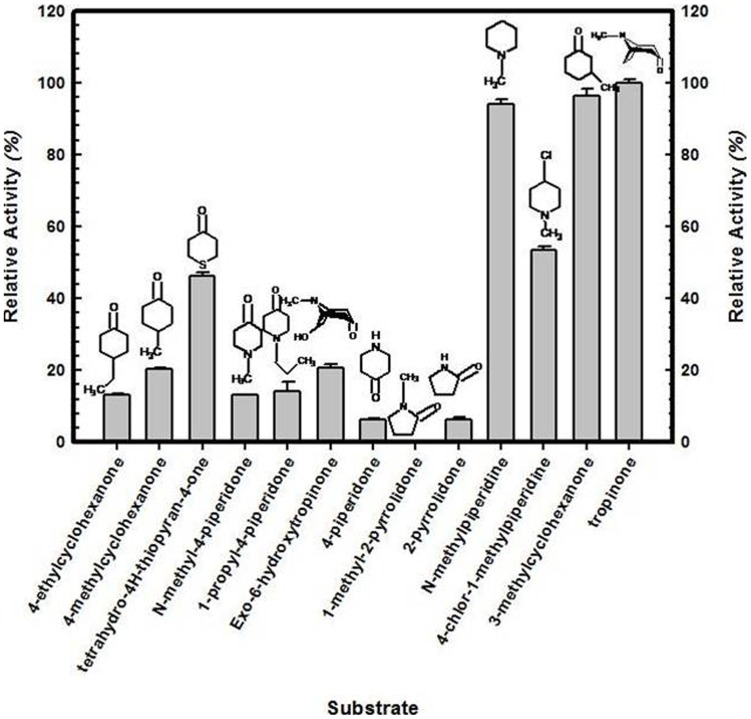
Comparative catalytic activity of tropine forming tropinone reductase of *W. somnifera* (WsTR-I) with different substrate analogues (at 5 mM concentration). Activity was measured relative to tropinone.

Several ketones and alcohols were assessed for their acceptance as substrate by the enzyme, respectively, in forward (NADPH-dependent) and reverse reaction (NADP^+^-dependent). Among the alternate substrates tested for forward reaction, all but one (1-methyl-2-pyrrolidone) were acceptable for the enzymatic reduction (Figure 7). Among them, the substrate analogues, 3-methylcyclohexanone and *N-*methylpiperidine were found to be reduced most efficiently (at par with tropinone) as discerned from their high affinity for the enzyme (Figure S4). Comparison of estimates of catalytic efficiency (K_cat_/K_m_) of the enzyme revealed it to be highest; for tropinone among the substrates (Table 2). Broader substrate specificity of WsTR-I resembles to that of TRs from other plant species [35], [41]. However, comparatively efficient catalysis of reduction of cyclic ketones (3-methylcyclohexanone and *N-*methylpiperidine), observed in this study for WsTR-I has not been reported earlier. For the reverse reaction, none of the tested alcohol worked (Figure S5). NADPH and NADP^+^, the cofactors for forward and reverse reaction, respectively, could not be replaced by NADH and NAD^+^. Thus, the enzyme had absolute specificity for NADPH/NADP^+^. With respect to reverse reaction, the cofactor's (NADP^+^) K_cat_/K_m_ value was almost same as for cofactor (NADPH) of forward reaction, whereas the value for reverse reaction substrate (tropine) was lower by >500 fold. This observation re-emphasizes that physiologically, the metabolic step may essentially operate in forward direction *in planta*.

#### Other effectors of WsTR-I activity

The measurement of enzyme activity in the presence of cations (K^+^, Mn^++^, Li^+^, NH_4_^+^, Mg^++^, Na^+^ and Co^++^, 5 mM each) revealed that, except slight enhancement of enzyme activity by Mg^++^ and Na^+^, none of these cations affected the activity significantly (*data not shown*).

#### Molecular evolution of WsTR-I

**Figure 8:**
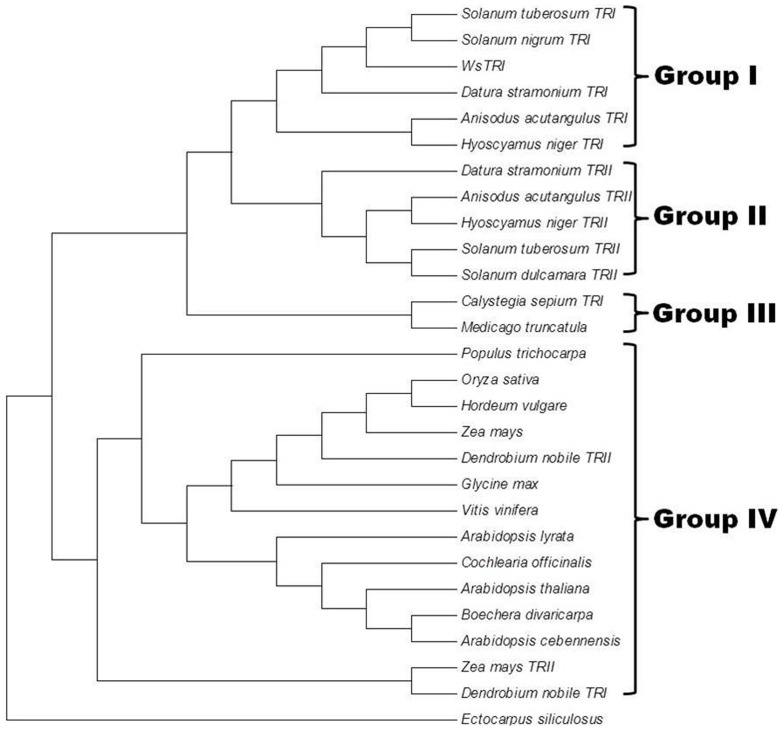
Phylogenetic relationship of tropine forming tropinone reductase of *W. somnifera* (WsTR-I) with other plant TRs. The tree was constructed with maximum likelihood method MEGA5 using sequences of *Solanum tuberosum* TRI (CAC34420.1), *Solanum nigrum* TRI (CAQ19734.1), *Datura stramonium* TRI (AAA33281.1), *Anisodus acutangulus* TRI (ACB71202.1), *Hyoscyamus niger* TRI (BAA85844.1), *Datura stramonium* TRII (AAA33282.1), *Anisodus acutangulus* TRII (ACB71203.1), *Hyoscyamus niger* TRII (AAB09776.1), *Solanum tuberosum* TRII (CAB52307.1), *Solanum dulcamara* TRII (CAQ19732.1), *Calystegia sepium* TRI (CAD20555.1), *Medicago truncatula* (ACJ84726.1), *Populus trichocarpa* (EEE95485.1), *Oryza sativa* (ABF95192.1), *Hordeum vulgare* (BAJ87177.1), *Zea mays* (ACG34080.1), *Dendrobium nobile* TRI (AFD23287.1), *Glycine max* (XP_003552795.1), *Vitis vinifera* (XP_002282554.1), *Arabidopsis lyrata* (ABW81053.1), *Cochlearia officinalis* (CAO02390.1), *Arabidopsis thaliana* (AAM10204.1), *Boechera divaricarpa* (ABW74581.1), *Arabidopsis cebennensis* (ABW81184.1), *Zea mays* TRII (NP_001147765.1), *Dendrobium nobile* TRII (AFD23289.1), *Ectocarpus siliculosus* (CBJ29615.1) along with the WsTR-I sequence.

At amino acid level, WsTR-I showed a high degree of homology to TRs of other Solanaceae plants. Therefore, it was appealing to examine its evolutionary position in the phylogeny of various TRs. An un-rooted phylogenetic tree was constructed by maximum likelihood method based on Jones-Taylor-Thornton model and analysed by bootstrap tool of MEGA5 programme (Figure 8). The analysis revealed that WsTR-I, SnTR-I, AaTR-I, HnTR-I, DsTR-I and StTR-I clustered together (Group I), whereas AaTR-II, HnTR-II, DsTR-II, SdTR-II and StTR-II grouped together into another cluster (Group II). Interestingly, CsTR-I formed a separate cluster along with putative TR from *Medicago sativa* (Group III). Putative TR or short chain dehydrogenase/reductase like proteins from plants that do not produce tropane alkaloids, such as PtTR, AtPTR and VvPTR, constituted the separate cluster (Group IV) along with CoTR from *Cochlaeria officinalis*, a cruciferous plant known to produce esters of tropine. Adding to its heterogeneity, the recently characterized TRs from *Dendrobium nobile* also joined this cluster (Group IV). CoTR and DnTRs are the only members of Group IV that have been characterized functionally. Interestingly, their characteristics are quite different from the members of Solanales' TRs characterized biochemically so far. It is considered that the two types of TRs using the same substrate and delivering products of different stereo-specificities are short-chain dehydrogenases/reductases (SDRs) that evolved from a common ancestor following gene duplication and mutational events [38]. CoTR had been reported as a nonspecific TR reducing tropinone into both stereo-isomeric alcohols i.e. tropine and pseudotropine. Thus, it holds both TR-I and TR-II activities, contrary to other TRs that are either TR-I or TR-II. These observations suggest a divergent evolution of TRs in Brassicaceae [48]. It is plausible to speculate that such a general TR could be a progenitor to evolve the specialized TRs (TR-I and TR-II) to ensure that only the desired stereo-specific form (tropine or pseudotropine) of the metabolite are produced, as physiologically called for in the target tissue. Similar evolutionary relationship was deduced in recent studies [49]. Nevertheless, while our manuscript was being reviewed, Cheng et al. [50] reported a tropinone reductase like gene (*DnTR-II*) from an orchid (*Dendrobium nobile*). It could not catalyze tropinone reduction at all, but its site directed mutagenesis at a single amino acid residue (Tyr^201^ to Val) in the substrate binding pocket of the enzyme enabled it with the catalytic capability. These studies conclude that substrate specificity of TRs may be determined by a few amino acids in substrate specific pocket and may not be related to phylogenetic branch [50]. Placement of different species in the phylogenetic tree generated here positioned WsTR-1 from *W. somnifera* close to the members of genus *Solanum* with maximal proximity to *S. nigrum* and *S. tuberosum*, congruent to phylogenetic analysis of Solanaceae carried out by Wink [51], Further, the members from Solanaceae plants known to produce tropane alkaloids clustered as a single polyphyletic group, farther from the non-producers like *Arabidopsis* and members of Poaceae, corroborating the distinctive significance of tropane alkaloids and TRs as new taxonomical markers.

#### Homodimeric nature of catalytically active WsTR-I

Molecular mass of catalytically active form of the recombinant WsTR-I protein, as assayed by size exclusion chromatography of the recombinant enzyme was compared with its polypeptide (subunit) molecular mass determined by SDS-PAGE (Figure 3) or theoretically computed for translated product of the complete cDNA. This comparison was aimed at determining the level of multiplicity of the constituent polypeptide in the catalytically active enzyme. It may have a reflection of structural features of the *in vivo* functional enzyme. The native molecular mass of the *WsTR-I* enzyme was estimated to be about 60 kDa, as determined by the plot of K_d_ against log_10_ molecular weight on chromatography through a calibrated column (with size marker proteins) (Figure S6). Whereas, mass of polypeptide, as computed for translated protein product of the cloned *WsTR-I* gene was 29.41 kDa. Therefore, clearly the catalytically active WsTR-I was a homo-dimer. It corroborates the singular other report on the plant tropinone reductase in this regard [44]. Further, in this biochemical perspective, relatively much fewer members of SDR family like TRs and (-)-isopiperitenol dehydrogenase (ISPD) appear to be multimeric in nature [52]. In fact, size of these dimeric enzymes (∼60 kDa) of SDR family brings them almost close to the size of general dehydrogenases. Thus, SDRs are sub-classifiable as short-chain monomeric dehydrogenases (SMoDR) and short-chain multimeric dehydrogenases/reductases (SMuDR).

### Homology Based Tri-Dimensional Molecular Model of WsTR-I

**Figure 9:**
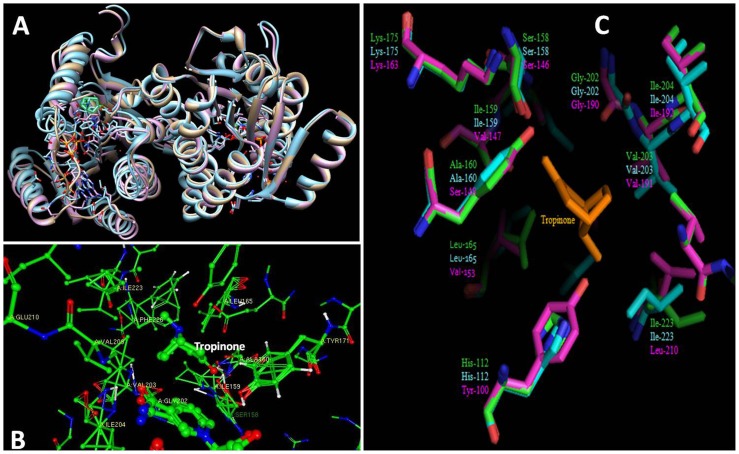
Homology based 3D model of tropine forming tropinone reductase of *W. somnifera* (WsTR-I). A, WsTR-I superimposed on to DsTR-I and DsTR-II. The model was constructed on Swiss-model workspace taking DsTRI (1ae1.pdb) as a template. Comparative representation was performed by UCSF Chimera package. WsTR-I, DsTR-I and DsTR-II are depicted as gray, purple and blue, respectively. NADPH and tropinone are visible in cleft of active site; B, Tropinone binding pocket of WsTR-I. A model was prepared by alignment of WsTR-I, DsTR-I and DsTR-II following energy minimization in Swiss-PDBviewer. Tropinone binding site was visualized by Ligand Explorer. Amino acids close to tropinone are labeled; C, Three dimensional (3-D) models of WsTR-I, DsTR-I and DsTR-II were aligned and analyzed in Pymol. Residues are lebelled in green (WsTR-I), cyan (DsTR-I) and magenta (DsTR-II). Tropinone is shown in orange.

A tri-dimensional (3D) molecular model of WsTR-I was built considering residues 16 to 273, showing sequence identity of 92.63% to the template structure of DsTR-I (1ae1.pdb). A model of dimeric protein was constructed by taking DsTR-I chain A (1ae1A.pdb) as additional chain and including NADP^+^ as ligand. The predicted model showed the typical tropinone reductase folding pattern of WsTR-I when compared to DsTR-I (1ae1.pdb) and DsTR-II (1ipf.pdb) (Figure 9A). WsTR-I was perfectly superimposed onto DsTR-I, while spatial differences were visible between TR-I and TR-II that highlight the sequence variability among different TRs. Tropinone was transferred into the predicted model by alignment of WsTR-I with 1ae1.pdb and 1ipf.pdb in Swiss-PDB Viewer following energy minimization. Tropinone binding pocket was explored using Ligand Explorer. At least twelve residues including Ala^160^, Val^168^, Ile^223^ and Phe^226^, were found in close proximity to the tropinone (Figure 9B). These four amino acid residues had been described critical for the stereospecificity of the reaction [41] and their proximity to the tropinone further substantiate the observation. Two residues, Ser^158^ and Tyr^171^, of catalytic tetrad N-S-Y-K [33] were also positioned in the vicinity of tropinone endorsing their role in catalysis. All the twelve residues, located in tropinone binding cleft, were found conserved at analogous positions in different TR-Is. A comparison of active site environments of different TRs showed strong homology between WsTR-I and DsTR-I, while there was significant variation between TR-I and TR-II (Figure 9C). At least five residues of WsTR-I including His^112^, Ile^159^, Ala^160^, Leu^165^ and Ile^223^ were substituted by Tyr^100^, Val^147^, ser^148^, Val^153^ and Leu^210^, respectively in TR-II. In a similar very recent analysis, Cheng et al. [50] reported close proximity of His^112^, Ser^158^, Ile^159^, Val^168^, Tyr^171^, Ile^223^ and Phe^226^ in active centre of their model built for an orchid TR like gene.

### Tropane Alkaloid Biosynthesis in *W. somnifera*

Biosynthesis of prodigal amounts of tropane alkaloids is known to be a characteristic feature of a few Solanaceae genera like *Hyoscyamus, Duboisia, Atropa*, and *Datura*. Some of these species also form the source of some commercially important and advanced tropanes like hyoscyamine and scopolamine. Nevertheless, presence of a few early alkaloids of tropane and pseudotropane classes have been sparsely reported from *Withania* [30], [32]. But, tropane alkaloid biosynthetic pathway and their genes or functional genomics or biochemical aspects of specific metabolic steps or intermediates have remained unattended for the plant. To make a beginning in this direction, we have investigated presence of the earliest tropane alkaloid moiety, (*i.e*. tropine) as well as expression of the tropine forming tropinone reductase (*WsTR-I*) in different tissues/parts of *W. somnifera*.

#### Comparative expression of WsTR-I in different tissues of *W. somnifera*

**Figure 10:**
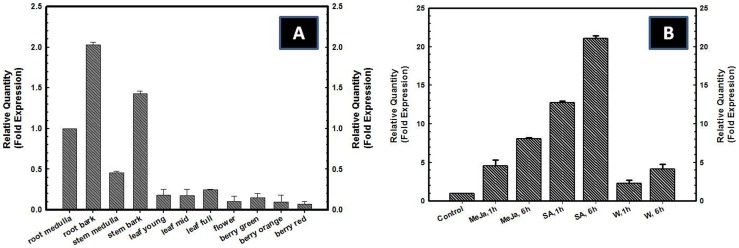
Real time PCR based comparative pattern of expression of tropine forming tropinone reductase gene (*WsTR-I*) in different tissues of *W.* * somnifera* (A) and real time PCR based assessment of effect signal molecules on WsTR-I expression (B). MeJa, 1 h, methyl jasmonate, 1 h treatment, MeJa, 6 h, methyl jasmonate, 6 h treatment, SA, 1 h, Salicylic acid, 1 h treatment, SA, 6 h, Salicylic acid, 6 h treatment, W, 1 h, Wounding, 1 h treatment, W, 6 h, Wounding, 6 h treatment.

To analyze the relative levels of expression of *WsTR-I* transcripts *in planta,* quantitative real time PCR (qRT-PCR) was performed using different plant tissues *i.e*. root bark, root medulla, stem bark, stem medulla, young leaves, mid-expansion leaves and mature leaves as well as flowers and berries of different developmental stages like immature (green), mature (orange) and ripe (red). Interestingly, *WsTR-I* transcripts were found to be expressed constitutively in all the tissues. However, the relative level of expression varied across the tissues/plant parts (Figure 10A). The gene was expressed at relatively higher levels in tissues such as root medulla, root bark, stem bark and stem medulla. Lowest level of expression was found in reproductive organs such as flowers and berries (Figure 10A). Ontogenically, the gene transcript levels increased in leaves with the progress of their growth and expansion and development (post-expansion maturation). In an opposite pattern, the gene expression declined slightly in berries with progress of growth and development (Figure 10A). Concept of constitutive expression of *WsTR-I* transcripts has become a developing concept in view of a few similar very recent reports [42], [49], [50], [53]. However, expression of a gene in alone in a tissue is not enough to infer its physiological operation and actual metabolic relevance *in planta*. It needs several other lines of evidences as provided ahead in this study.

#### Modulation of WsTRI gene expression by signal molecules and wounding

Exposure of leaf to methyl jasmonate (MeJa) and salicylic acid (SA) as well as tissue mechanical injury (wounding) up-regulated expression of the *WsTR-I* gene, as revealed by the results of qRT-PCR. Significant elevation in TR transcript levels was evident even during first hour after the application of MeJa, SA and wounding (Figure 10B). Levels were further elevated at 6 h. Effect of SA was most profound with ∼21 fold increase in the TR transcript level after 6 h of the treatment, while MeJa and wounding enhanced the expression by ∼11 and ∼4 fold, respectively. As these molecules also serve as stress signals, modulation of the gene expression by them, similar to wound/injury stress, may reflect that the TR may also participate in the physiological and metabolic mechanisms of stress response/management. Stress signalling molecules mediated enhancement of expression of TR-I is comparable to the few earlier observations on the gene from other plants [42], [49], [50].

Constitutive expression pattern of *TR-I* in *W. somnifera* prompted us to explore if this expression manifests independent *de novo* metabolic competence of these tissues to synthesize tropane alkaloids. The observed development and tissue-specific variations in levels of the gene expression were also impetuous to investigate this domain of tropane alkaloid metabolism. In plants that prodigally produce tropanes like *A. belladona*, *H. muticus*, *H. albus*, *Duboisia myoporides*, it is considered that roots are biosynthetic sites while shoots solely serve as the storage (sequestration) site/tissues. It is in synchrony to this that, in agrarian practice, only shoots are harvested from their crops and processed for formulations, tinctures and phytochemicals. Contrarily, in case of *W. somnifera*, as per traditional system of medicines, mainly roots are used for the preparation of pharmacological and nutraceutical products.

The phenomenon of separation of biosynthetic and sequestration sites for secondary metabolite at the level of root and shoot represents the widest partitioning of the two activities of secondary metabolism in plants. This notion for tropane alkaloids has been mainly based on reciprocal grafting experiments that began in 1940s [54], i.e. much before the tools of molecular analysis of gene expression were available and even before much of the pathway was known. Our observed pattern of TR gene expression failed to fit this classical notion of solely root restricted biosynthesis of tropane alkaloids. Nevertheless, mere detection of expression of the pathway gene in a plant organ, does not necessarily mean that the end products of the biosynthetic pathway are synthesized there. The inferred conceptual line of actual and independent biosynthesis of tropanes in aerial tissues needs corroborating evidences from *in vivo* experiments like radio-tracer experiments and detection of relevant intermediary metabolites of the pathway related to the enzyme. Therefore, this investigation was advanced to such metabolite analyses in aerial tissues and *in vivo* experiments on the plant.

#### Tropinone and tropine levels in different parts/tissues of *W. somnifera*

TLC separation of the alkaloid extracts from different tissues of the plant followed by chromo-detection with a modified Dragandorff's reagent revealed at least two spots in root bark, stem bark, young, mid- and fully- expanded leaves and flowers that corresponded to tropinone and tropine (data not shown). In tissues, such as root medulla, stem medulla and berries (immature, mature and ripe), presence of tropine was clearly visible. TLC profile of these tissues was further validated by gas chromatographic quantification. Peaks corresponding to standard tropinone and tropine were detected in all the tissues except in immature and mature berries. Tropinone levels were comparatively low in all the tissues studied, ranging from 2.32 µg g^−1^ dry weight (DW) in stem medulla to 91.61 µg g^−1^ DW in flowers (Table 3). Highest level of tropine was detected in stem medulla (2,096.85 µg g^−1^ DW), while lowest was in mature berries (38.73 µg g^−1^ DW) (Table 3). Reduced levels of tropinone and tropine in berries matched with the lower expression of *WsTR-I* transcripts therein (Figure 10A), suggesting that berries are relatively poor sites of tropane alkaloid biosynthesis.

**Table 3:** Gas chromatography-based quantification of tropinone and tropine in different tissues of *Withania somnifera*.

Tissue	Tropinone (µg g^−1^ DW#)	Tropine (µg g^−1^ DW#)	Tropine to tropinone ratio
Root medulla	36.52±1.53	2096.85±10.98	57.41
Root bark	39.92±2.84	1966.1±8.87	49.25
Stem medulla	2.32±0.09	1801.19±12.64	776.375
Stem bark	37.88±2.9	1582.0±14.97	41.76
Leaf young	44.0±1.64	463.04±2.08	10.52
Leaf mid expansion	30.38±0.98	758.33±1.47	24.96
Leaf full expansion	10.38±0.74	860.38±5.64	82.88
Flower	91.61±4.86	758.1±3.65	8.27
Berry immature	Not detected	39.44±2.9	-
Berry mature	Not detected	38.73±0.9	-
Berry ripe	3.15±0.18	79.39±3.26	25.20

# DW: Dry weight.

Overall, the observed presence of the TR-I substrate as well as product in quite significant concentrations in the aerial parts of the plant is corroborative to the gene-expression profile led line of inference of the possibility of actual biosynthesis of tropane alkaloids in the aerial parts of the plant. A concurrent analysis of the pathway genes of tropane metabolism in *Hyoscyamus senecionis* [53] indicates to the similar hypothesis of *de novo* synthesis of tropanes in aerial tissues.

#### *In vivo* incorporation of label from U-[14C]-sucrose into tropanes by orphan shoots (twigs)

*WsTR-I* gene expression and relevant metabolite levels observations inferred hypothesis of *de novo* biogenesis of tropanes. This was further validated by radiotracer studies on the plant shoots (twigs without roots) using U-[^14^C]-sucrose. After a chase period of 72 h, the label incorporation from the sucrose to tropinone and tropine was chased both qualitatively (radio-TLC phospho-imaging) and quantitatively (liquid scintillation counting). Phospho-images of the TLC run of the leaf and stem incorporated extracts showed the fluorescence at run positions (R_f_) corresponding the co-chromatographed tropane standards (tropinone, tropine and pseudotropine). Additionally these spots were detected chromogenically after phosphor-imaging of the plate (Figure S7). The quantitative levels of the ^14^C-label incorporation (*de novo* biogenesis) of tropinone, tropine and pseudotropine in stem and leaf is presented in Table 4. While *de novo* biosynthesis of the three tropane moieties was found in leaf as well as stem, the observed higher level of the label incorporation into tropine than tropinone correlated with their relative pattern of accumulation observed in the assays of the metabolites in the tissue (Table 3). This is understandable as tropinone is a metabolic intermediate while tropine is a putative end product of tropane metabolism in *W. Somnifera*. The observed higher tropine to tropinone ratio of accumulation (long-term) *in planta* (Table 3), as compared to that noted in short-term *in situ* biogenesis study (Table 4) can also be inferred in the above metabolic inter-relationship perspective, besides due to any contribution of the tropine translocated from roots. The almost two fold higher ratio of tropine to tropinone biogenesis than pseudotropine to tropinone (Table 4) reflects, major flux of the substrate (tropinone) towards WsTR-I catalysed tropine production. Also, higher biogenetic ratios in stem than in leaf (Table 4) coincided with the observed relatively higher level of the gene expression pattern of *WsTR-I* in the tissue.

**Table 4:** Quantitative results of incorporation of radiolabel (^14^C) from U-[^14^C]-sucrose into tropanes (tropine and pseudotropine) by rootless shoots (twigs) of *W. somnifera*.

Tissue	Tropinone (CPM/ 100 g Tissue)	Tropine (CPM/ 100 g Tissue)	Pseudotropine (CPM/ 100 g Tissue)	Tropine to tropinone ratio	Pseudotropine to tropinone ratio
Stem	6960±65	30112±125	13990±67	4.3	2.0
Leaves	10652±115	23010±83	13704±95	2.1	1.3

Thus, results of the study on aerial tissues with respect to (i) presence of transcript of *WsTR-I* gene and profound (many fold) up-regulation in response to external stimuli such as MeJa, SA, injury, (ii) detection of the substrate (tropinone) and product (tropine) of the enzyme catalysed reaction, (iii) actual synthesis of tropinone and tropine from primary precursor (sucrose); unambiguously elucidate inherent metabolic competence of aerial tissues to independent synthesis of tropane alkaloids *de novo*, at least in *W. somnifera*. It also implies that the role of roots, in accounting to the shoots accumulated tropane alkaloids, is not exclusive. Conceptually, it is a deviation from the inferences of grafting experiments conducted on other plants (*Datura*/*Hyoscyamus*/*Atropa*) that tropane alkaloids are synthesized in roots and transported to aerial parts [54]. The reported TR-I catalytic activity, detectable in roots alone [35] and immuno-localization experiments [45], [55] emphatic to the presence of TR proteins in below ground organs of these plants have been supportive to the theory of acropetal transport of tropane alkaloids in them. Nevertheless, operation of similar translocation of tropane alkaloids in *W. somnifera* as a physiological activity concurrent to *in situ* synthesis is still a possibility to be examined. Moreover, relative contribution of root/shoot in tropane alkaloid biosynthesis could also be quite different in these plants (*Datura*/*Hyoscyamus*/*Atropa*) than in the evolutionarily distant *W. somnifera*. But a very recent report on a *Hyoscyamus* species (*H. senescionis*) elucidates aerial tissue expression of scopolamine biosynthetic genes as well as some of the upstream genes of the pathway in the plant [53]. Also, recently, a gene encoding tropine forming tropinone reductase was isolated from the leaves of *W. coagulans* by our group [56]. While this manuscript was under revision, a report has appeared on an aerial tissue expressed gene of an orchid (*D. nobile*) encoding tropinone reductase like protein that was catalytically unable to use tropinone as a substrate in its native form but could be enabled for it by site-directed mutagenesis at a single residue in its active site [50]. These could be indicative lines of broader operation of the phenomenon across the plant species and families.

To our knowledge, present study constitutes the first report providing integrated physiological, biochemical and molecular evidences and thereby unambiguously elucidating independent *de novo* tropane alkaloid biosynthesis in the aerial parts of the plant. These new lines of evidences underpin the need of investigational revisits on tropane alkaloid metabolism in other plants to elaborate the postulation for a broader renewed synthesis of the subject.

## Conclusions

Tropine forming tropinone reductase of *Withania somnifera* gene codes for a polypeptide (∼30 kDa) that was highly homologous to other members of the short chain dehydrogenases/reductases (SDR) family. The catalytically active enzyme was 60 kDa homodimeric protein. The enzyme had catalytic characteristic and substrate specificity similar to other reported TR-Is. Interestingly, the gene was considerably expressed in most of the plant parts rather than remaining only restricted to roots reported for other species. *WsTR-I* was manifold up-regulated by external stimuli in aerial tissues. This, together with profile of metabolites (tropinone and tropine) and findings of *in vivo* radio-tracer studies, unambiguously elucidated that several parts of *Withania somnifera* plant possess inherent capability to independently synthesize tropane alkaloids *de novo*. The observed novelties of the enzyme have been discussed with respect to their physiological overtones including in relation to current concept of acropetal transport of tropanes in Solanaceae. To our knowledge, this pertains to be the first report as concerns integrated investigation of tropane alkaloids with respect to physiological, biochemical and molecular aspects of TR catalysed step of the pathway in the plant. Some of the observed novelties in this study call for a new scope of fundamentals of alkaloids for this medicinal plant that is gaining growing validations for its traditional pharmacological activities.

## Supporting Information

Figures S1 **Thermostabilty of WsTR-I.** A) Effect of temperature on WsTR-I: WsTR-I protein was incubated for 30 min at different temperatures. Untreated protein was taken as positive control. Activity was assessed as described. In negative control protein was excluded. B) Effect of additives on thermostability of WsTR-I: Protein was incubated at different temperatures in presence of 2-mercaptoethanol (5 mM), glycerol (20%) and sucrose (20%) and activity was measured as described in text.
(TIF)

Figures S2 **Substrate saturation curves for forward reactions.** The assay mixtures were prepared by sequentially increasing concentration of tropinone or NADPH.
(TIF)

Figures S3 **Substrate saturation curves for reverse reactions.** The assay mixtures were prepared by sequentially increasing concentration of tropine or NADP^+^.
(TIF)

Figures S4 **Substrate saturation curves for substrate analogues.** The assay mixtures were prepared by sequentially increasing concentration of 3-methylcyclohexanone or *N-*methylpiperidine.
(TIF)

Figures S5 **Alcohols tested as substrates for the reverse reaction catalysis by WsTR-I.**
(TIF)

Figures S6 **Estimation of molecular mass of catalytic active WsTR-I protein.** Protein was chromatographed in a column packed with Sephadex G-200 resin along with protein molecular weight standards. K_d_ (WsTR-I) was plotted against log 10 Molecular Weight of known proteins. Molecular mass of WsTR-I was estimated to be 60 kDa by the deduced log_10_ of molecular weight.
(TIF)

Figures S7 **Thin layer chromatography of radiolabeled extracts of leaves and stem.** A) Iodine stained TLC, 1, tropinone standard, 2, tropine standard, 3, pseudotropine standard; B, Radiograph of TLC as visualized by phosphoimager; C) Chromo-staining of tropane standards by Dragandorff reagent, 1, tropinone standard, 2, tropine standard, 3, pseudotropine standard.
(TIF)
