# Supplementary figures and images for: Tropine Forming Tropinone Reductase Gene from Withania somnifera (Ashwagandha): Biochemical Characteristics of the Recombinant Enzyme and Novel Physiological Overtones of Tissue-Wide Gene Expression Patterns
Source: PLoS One. 2013 Sep 25;8(9):e74777. doi: 10.1371/journal.pone.0074777 (PMC3783447; doi:10.1371/journal.pone.0074777)

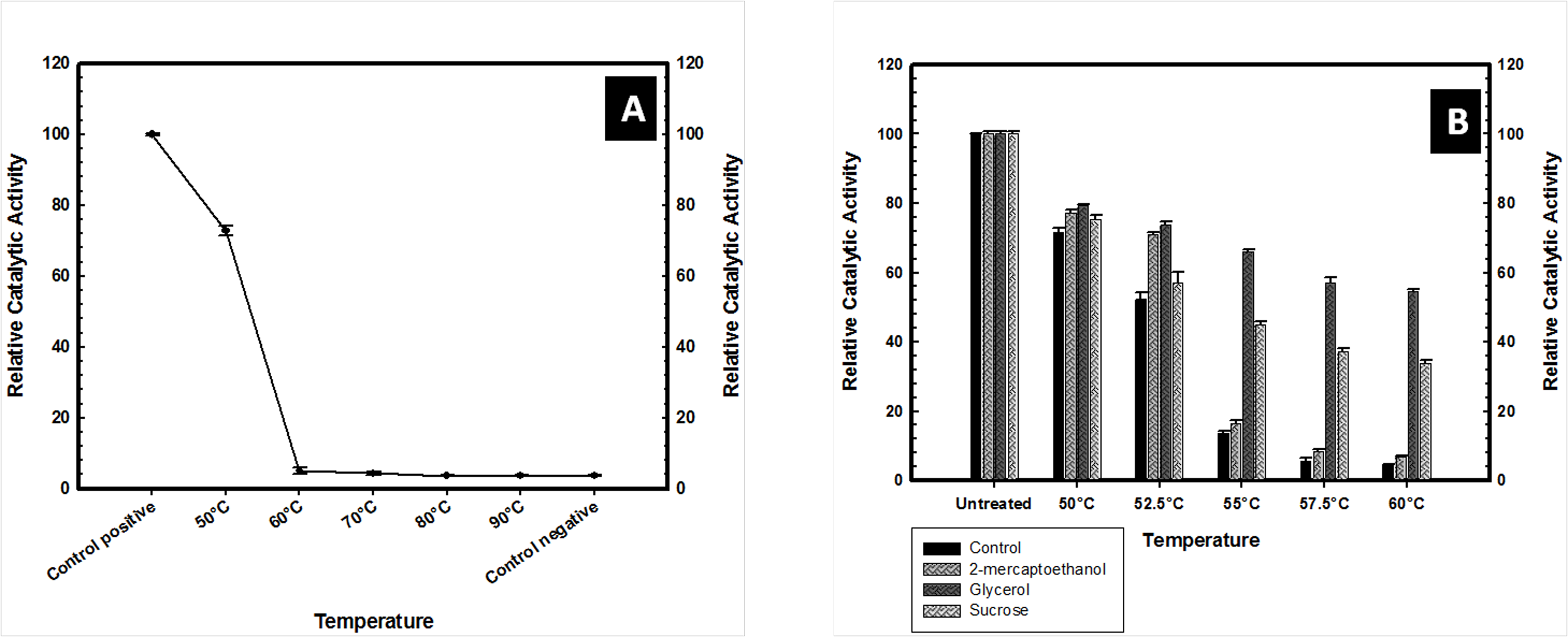

Supplement: Figures S1 — Thermostabilty of WsTR-I. A) Effect of temperature on WsTR-I: WsTR-I protein was incubated for 30 min at different temperatures. Untreated protein was taken as positive control. Activity was assessed as described. In negative control protein was excluded. B) Effect of additives on thermostability of WsTR-I: Protein was incubated at different temperatures in presence of 2-mercaptoethanol (5 mM), glycerol (20%) and sucrose (20%) and activity was measured as described in text. (TIF) [file pone.0074777.s001.tif]

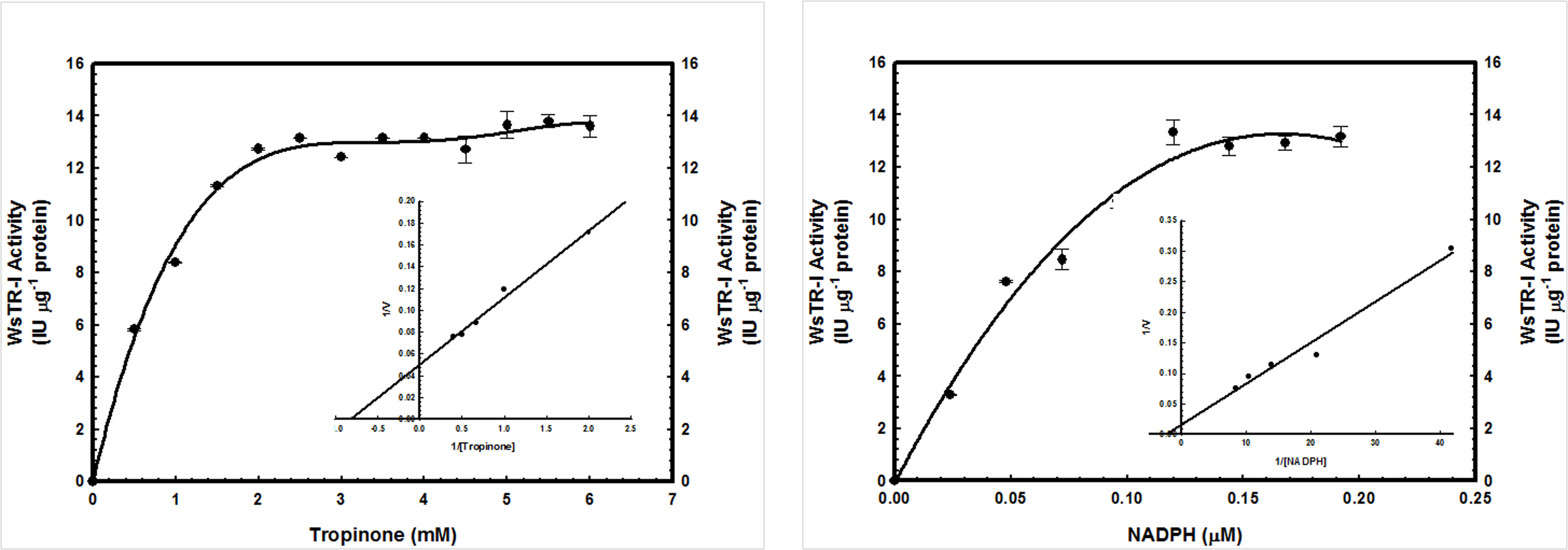

Supplement: Figures S2 — Substrate saturation curves for forward reactions. The assay mixtures were prepared by sequentially increasing concentration of tropinone or NADPH. (TIF) [file pone.0074777.s002.tif]

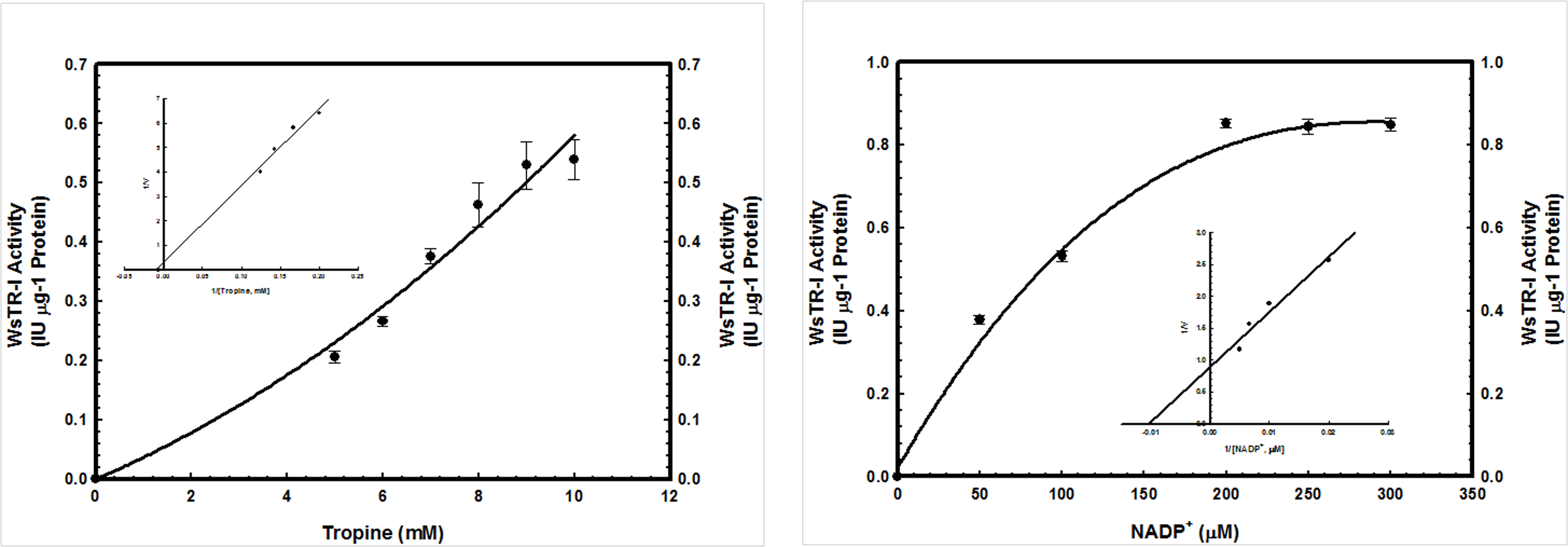

Supplement: Figures S3 — Substrate saturation curves for reverse reactions. The assay mixtures were prepared by sequentially increasing concentration of tropine or NADP+. (TIF) [file pone.0074777.s003.tif]

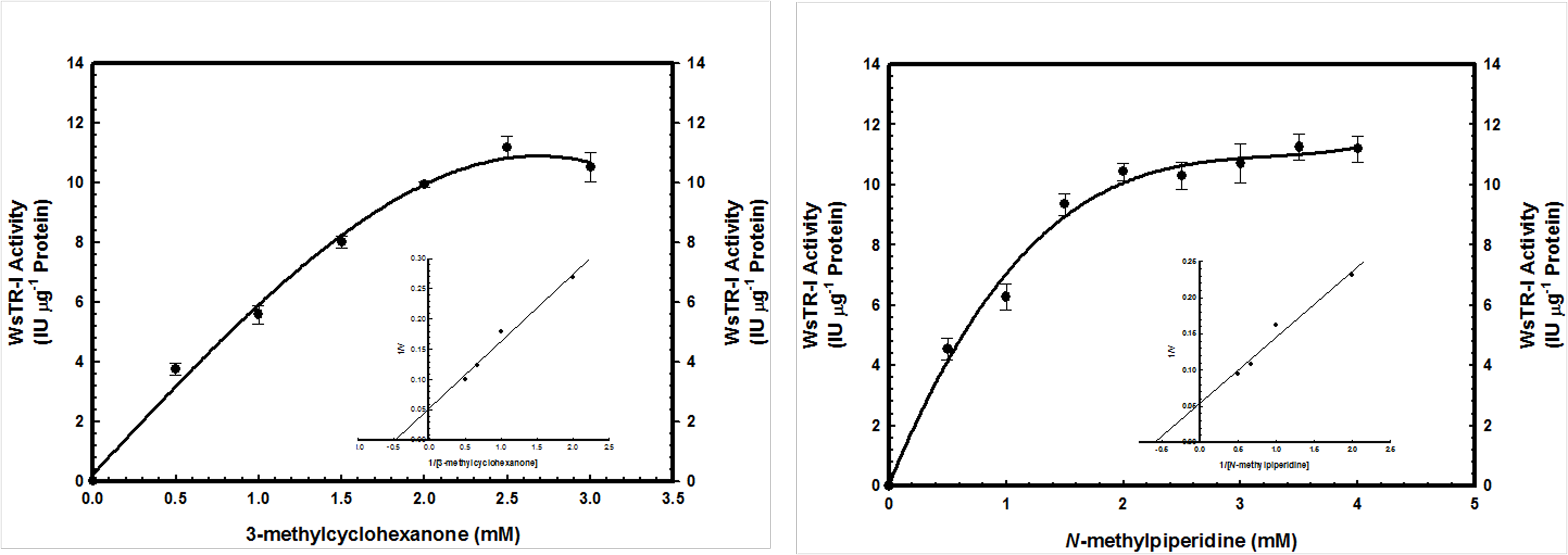

Supplement: Figures S4 — Substrate saturation curves for substrate analogues. The assay mixtures were prepared by sequentially increasing concentration of 3-methylcyclohexanone or N-methylpiperidine. (TIF) [file pone.0074777.s004.tif]

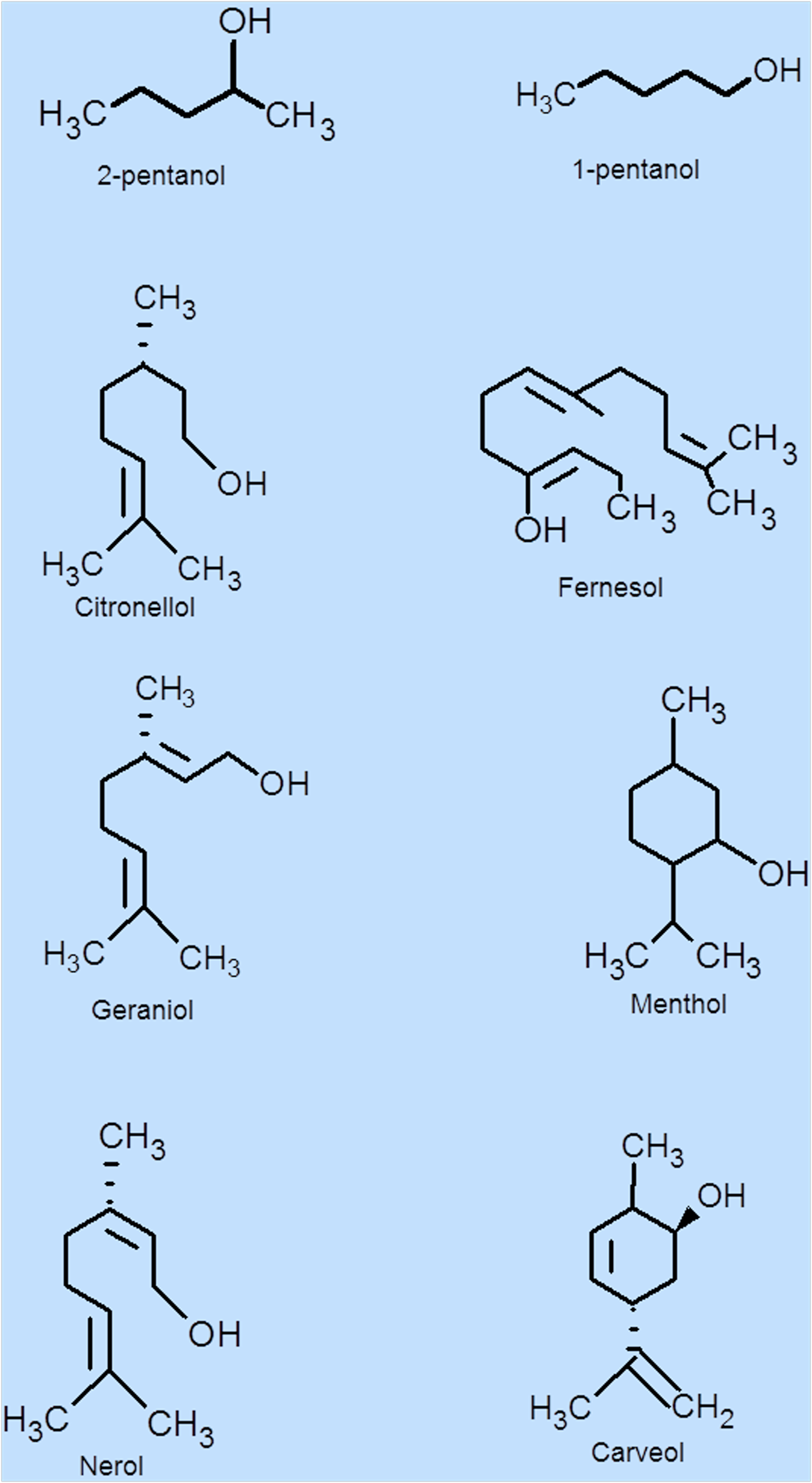

Supplement: Figures S5 — Alcohols tested as substrates for the reverse reaction catalysis by WsTR-I. (TIF) [file pone.0074777.s005.tif]

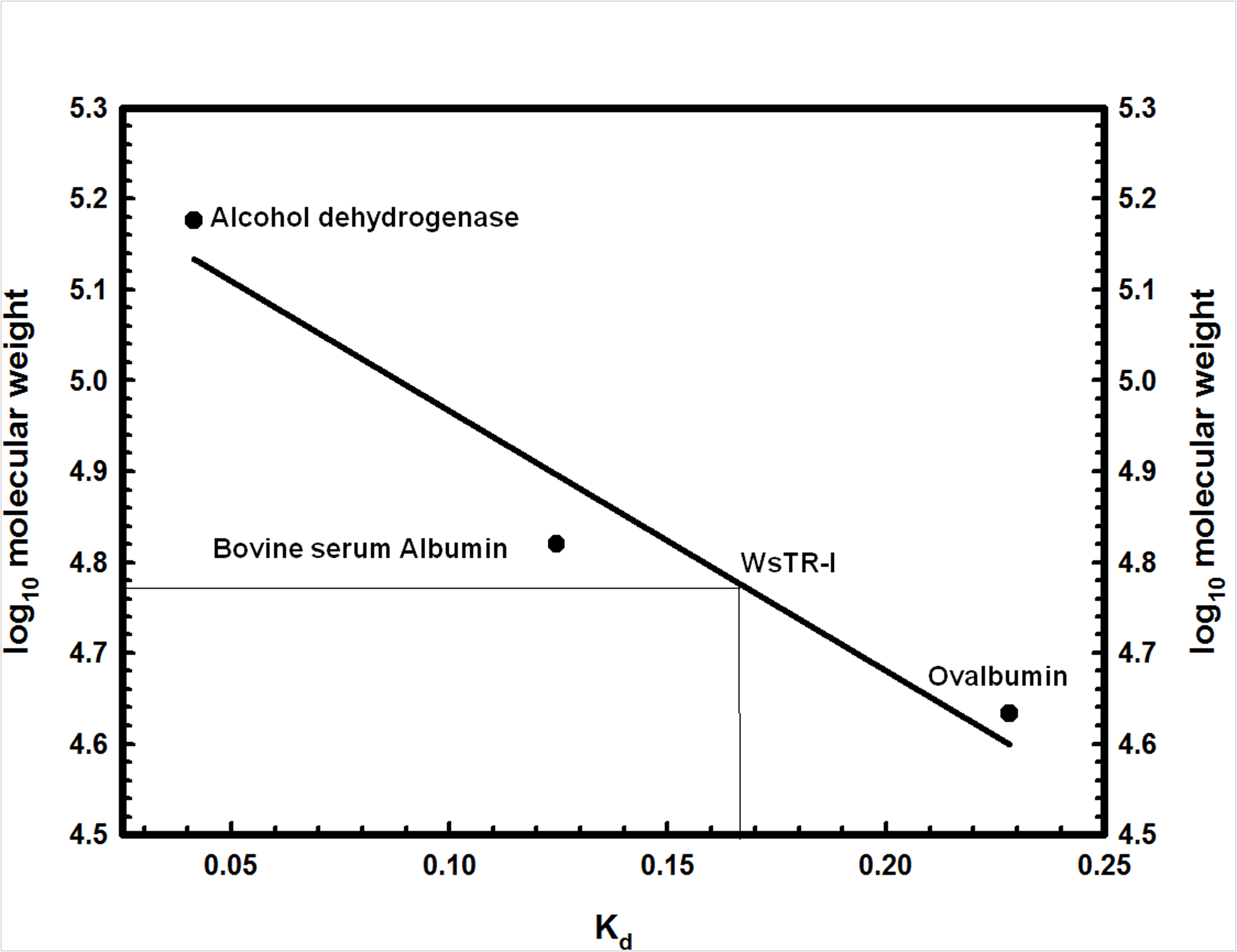

Supplement: Figures S6 — Estimation of molecular mass of catalytic active WsTR-I protein. Protein was chromatographed in a column packed with Sephadex G-200 resin along with protein molecular weight standards. Kd (WsTR-I) was plotted against log 10 Molecular Weight of known proteins. Molecular mass of WsTR-I was estimated to be 60 kDa by the deduced log10 of molecular weight. (TIF) [file pone.0074777.s006.tif]

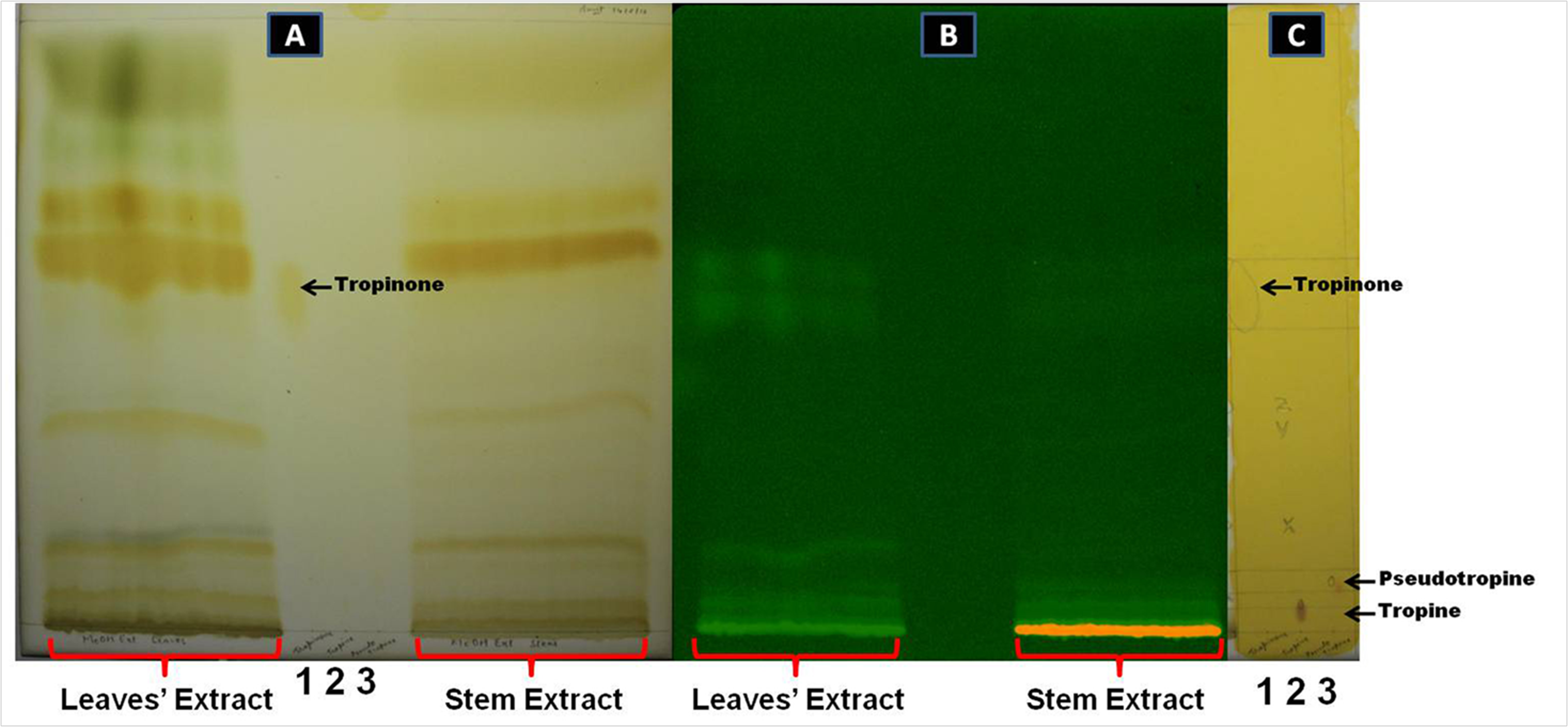

Supplement: Figures S7 — Thin layer chromatography of radiolabeled extracts of leaves and stem. A) Iodine stained TLC, 1, tropinone standard, 2, tropine standard, 3, pseudotropine standard; B, Radiograph of TLC as visualized by phosphoimager; C) Chromo-staining of tropane standards by Dragandorff reagent, 1, tropinone standard, 2, tropine standard, 3, pseudotropine standard. (TIF) [file pone.0074777.s007.tif]
